# Identification of Potential Biomarkers and Immune Features of Sepsis Using Bioinformatics Analysis

**DOI:** 10.1155/2020/3432587

**Published:** 2020-10-09

**Authors:** Fang-Chen Gong, Ran Ji, Yu-Ming Wang, Zhi-Tao Yang, Ying Chen, En-Qiang Mao, Er-Zhen Chen

**Affiliations:** Department of Emergency in Ruijin Hospital Affiliated to Shanghai Jiao Tong University School of Medicine, Shanghai, China

## Abstract

Sepsis remains a major global concern and is associated with high mortality and morbidity despite improvements in its management. Markers currently in use have shortcomings such as a lack of specificity and failures in the early detection of sepsis. In this study, we aimed to identify key genes involved in the molecular mechanisms of sepsis and search for potential new biomarkers and treatment targets for sepsis using bioinformatics analyses. Three datasets (GSE95233, GSE57065, and GSE28750) associated with sepsis were downloaded from the public functional genomics data repository Gene Expression Omnibus. Differentially expressed genes (DEGs) were identified using R packages (Affy and limma). Functional enrichment of the DEGs was analyzed with the DAVID database. Protein-protein interaction networks were derived using the STRING database and visualized using Cytoscape software. Potential biomarker genes were analyzed using receiver operating characteristic (ROC) curves in the R package (pROC). The three datasets included 156 whole blood RNA samples from 89 sepsis patients and 67 healthy controls. Between the two groups, 568 DEGs were identified, among which 315 were upregulated and 253 were downregulated in the septic group. These genes were enriched for pathways mainly involved in the innate immune response, T-cell biology, antigen presentation, and natural killer cell function. ROC analyses identified nine genes—LRG1, ELANE, TP53, LCK, TBX21, ZAP70, CD247, ITK, and FYN—as potential new biomarkers for sepsis. Real-time PCR confirmed that the expression of seven of these genes was in accordance with the microarray results. This study revealed imbalanced immune responses at the transcriptomic level during early sepsis and identified nine genes as potential biomarkers for sepsis.

## 1. Introduction

Sepsis is defined as a life-threatening organ dysfunction caused by a dysregulated host response to infection. Despite advances in critical care management over the past few years, sepsis is still associated with high mortality and morbidity worldwide [1]. It has been reported that sepsis causes 30 million episodes and 6 million deaths per year globally. However, according to the WHO, the data have missed incidences in the low- and middle-income countries, which means that the true burden arising from sepsis is far more serious. Therefore, the early diagnosis of sepsis is necessary to provide timely treatment. Markers currently in use, for example, CRP, PCT, and IL-6, have intrinsic shortcomings such as a lack of specificity and failures in the early detection of sepsis [2]. Many researchers are committed to exploring new biomarkers for sepsis. For example, studies have found that serum levels of presepsin, soluble urokinase plasminogen activator receptor, and soluble triggering receptor expressed on myeloid cell 1, as well as the expression of CD64, are upregulated among sepsis patients. Newly identified classes of biomarkers such as microRNAs, long noncoding RNAs, and the human microbiome are also arousing general interest [3]. Despite the increase in different potential biomarkers, such efforts have not yet yielded satisfactory results, which warrants further validation.

It is not surprising that a large proportion of sepsis biomarkers still focuses on the inflammatory part of this condition. Sepsis is characterized by disrupted inflammatory responses. It has been proposed that following a major inflammatory insult, there are simultaneous inflammatory and immunosuppressive responses [4]. Pattern recognition receptors such as TLR recognize and elicit inflammatory responses against pathogenic factors, for example, by triggering leukocyte and complement activation [5]. Concurrent immune cell function impairment (e.g., neutrophil defects) and T-cell apoptosis also occur, leading to immune suppression in patients with sepsis [6]. This could trigger secondary infections and undermine the immune system. However, the complex inflammatory responses during sepsis have not been fully elucidated.

Bioinformatics analysis offers an ideal way to screen large gene expression datasets to comprehensively understand the mechanisms underlying sepsis. In this study, we integrated three datasets and used a bioinformatics analysis approach to detect key genes and potential new biomarkers involved in sepsis. Molecular mechanisms underlying the inflammatory responses during sepsis were also explored to search for possible new treatment targets for sepsis.

## 2. Materials and Methods

### 2.1. Data Sources

The three gene expression datasets analyzed in this study were downloaded from the Gene Expression Omnibus (GEO) database (https://www.ncbi.nlm.nih.gov/geo/) and used to identify DEGs. GSE95233 [7], GSE57065 [8], and GSE28750 [9] were taken as representative datasets of patients with sepsis. For each dataset, data from day 1 of sample collection were used to analyze the gene expression based on the GPL570 platform (HG-U133_Plus_2). All data were freely available online, and this study did not involve any human or animal experiments.

### 2.2. Identification of DEGs

Background expression value correction and data normalization of the raw data were carried out using the Affy package in R (Affy, version 1.64.0). Subsequently, the Linear Models for Microarray Analysis R package (limma; version 3.42.2) was applied for differential expression analysis. Volcano plots were generated using Bioconductor (http://bioconductor.org/biocLite.R). DEGs were identified as those with a *t*-test value of *P* < 0.05 and a [logFC] > 1.5.

### 2.3. Functional and Pathway Enrichment Analyses

Gene Ontology (GO) analyses were used for the exploration of functional roles of gene sets, while KEGG analyses were used to classify the pathways in which such genes might function. For comprehensive functional annotation, GO and KEGG analyses of the identified DEGs were conducted using the DAVID tool (https://david.ncifcrf.gov/). A false discovery rate (FDR) < 0.05 in both GO and KEGG analyses was set as the threshold for significant enrichment.

### 2.4. Protein-Protein Interaction (PPI) Network Construction and Hub Gene Analysis

DEGs were uploaded to Search Tool for the Retrieval of Interacting Genes (STRING, https://string-db.org/) to analyze interactions among the proteins encoded by the identified DEGs. Results with a minimum interaction score of 0.4 were visualized using Cytoscape. The PPI network for hub genes was computed with the maximal clique centrality (MCC) method and CytoHubba. Based on the MCODE plugin, the PPI network was divided into two clusters (clusters A and B).

### 2.5. Receiver Operating Characteristic (ROC) Curve Analysis

ROC curve analyses to determine the specificity, sensitivity, likelihood ratios, positive predictive values, and negative predictive values for all possible thresholds of the ROC curve were performed using the R package (pROC, version 1.16.2). The diagnostic values of the genes were predicted based on the ROC curve analysis.

### 2.6. Patient Enrollment

Patients diagnosed with sepsis in the emergency department of Shanghai Ruijin Hospital from July 31, 2020, to August 21, 2020, were enrolled. This part of the study was approved by the Ethics Committee of Ruijin Hospital (No. 2017119). Enrollment criteria were as follows: (1) age: 18–90 years old, (2) patients met sepsis 3.0 sepsis diagnostic criteria, and (3) hospital stay > 24 h. Exclusion criteria were as follows: (1) discharged or died within 24 h after admission, (2) participated in other clinical research, (3) needed emergency surgery after admission, (4) had a malignant tumor, (5) pregnant or lactating, and (6) lacked necessary clinical data.

### 2.7. Quantitative Real-Time PCR

RNA was extracted from whole blood using TRIzol reagent (12183-555, Invitrogen) following the manufacturer's instructions. cDNA was synthesized with a SuperScript™ III First-Strand Synthesis SuperMix for qRT-PCR (11752-050, Invitrogen). Power SYBR^®^ Green PCR Master Mix (4367659, Applied Biosystems) was used for qRT-PCR to analyze mRNA expression. GAPDH was used as an internal control, and the relative mRNA expression levels were calculated using the 2^–*ΔΔ*CT^ method. The primer pairs used in the experiments are listed in data S1.

## 3. Results

### 3.1. Identification of DEGs

**Figure 1:**
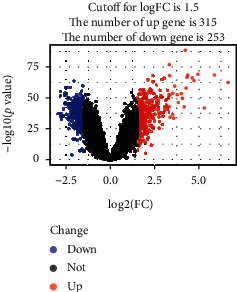
Identification of DEGs from three datasets using volcano plot analysis. Statistically significant DEGs were identified as those with a *t*-test *P* value < 0.05 and a [logFC] > 1.5. The blue dots represent downregulated DEGs, while the red dots represent upregulated DEGs.

Datasets GSE57065, GSE95233, and GSE28750 were downloaded from the GEO database and analyzed using R packages (Affy, limma, and ggplot2). Volcano plots were generated to visualize fold changes of the DEGs. Of the 568 DEGs evaluated, 315 were upregulated and 253 were downregulated in the sepsis group (Figure 1).

### 3.2. Functional Enrichment of DEGs

**Figure 2:**
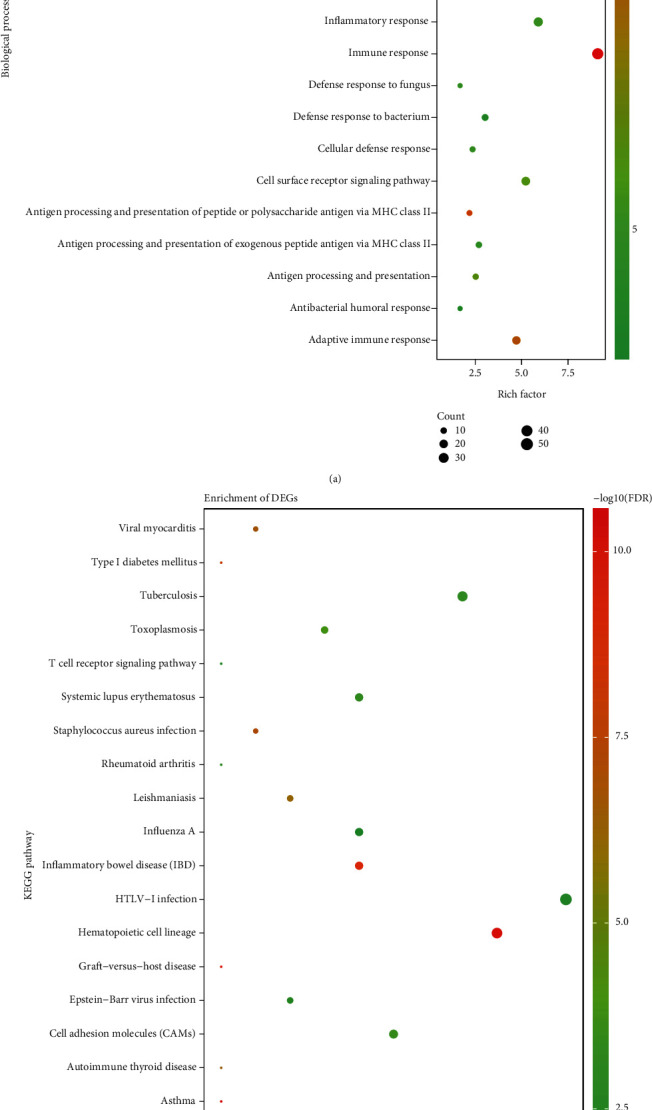
GO and KEGG enrichment analyses of DEGs. (a) Shows the results of biological process terms enriched by GO analysis. (b) Shows the enriched pathway by KEGG analysis. The colored dots represent the FDR for that term, with red representing greater significance. The size of the dots represents the number of involved genes. The rich factor represents the proportion of enriched genes for each term.

Enrichment analysis techniques extract biological information from a set of genes or proteins. To identify key genes related to sepsis, gene functions were annotated using the DAVID online software database. GO annotation analysis showed enrichment of DEGs involved in inflammatory responses such as the innate response, T-cell receptor pathway, and antigen processing and presentation (Figure 2(a)). KEGG pathway analysis showed that the genes involved in sepsis were associated with different infections such as influenza, tuberculosis, and HTLV-1, further linking inflammation with sepsis (Figure 2(b)).

### 3.3. PPI Network and Hub Genes

**Figure 3:**
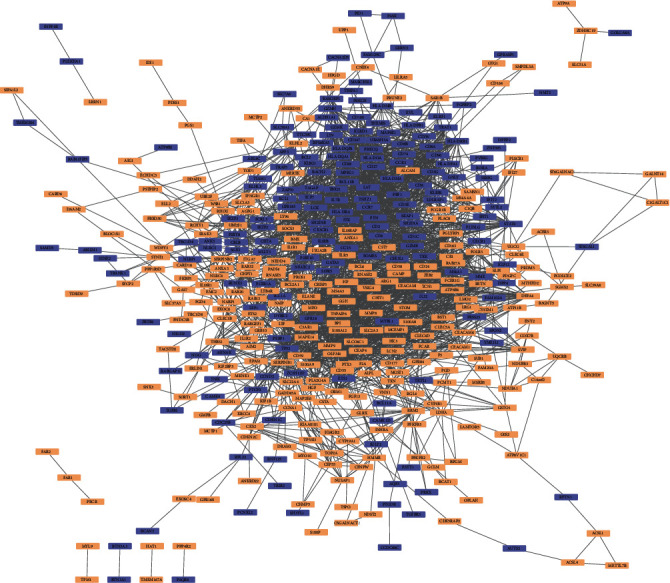
PPI network complex of DEGs. 568 DEGs with 443 nodes and 2470 edges were displayed by the PPI network, with upregulated genes shown in light orange and downregulated genes shown in bluish violet.

**Figure 4:**
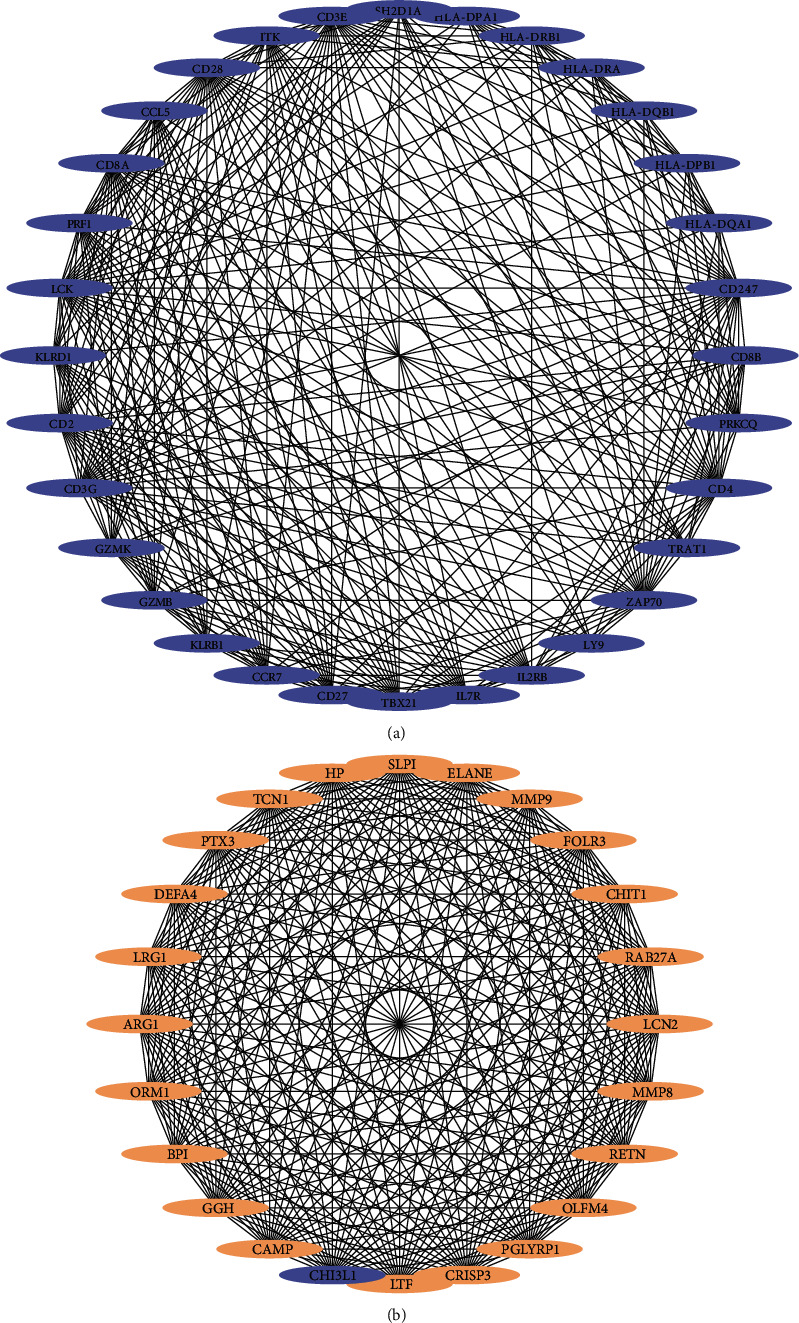
Clusters A and B identified by network analysis. The PPI network is divided into 2 clusters by the MCODE plugin. Light orange and bluish violet represent upregulated and downregulated genes separately.

To explore the key genes involved in sepsis, a PPI network with 443 nodes and 2470 edges was built for the 568 DEGs (Figure 3).  Table 1(a) shows the top 20 genes with the highest degree rank, whereas the top 20 genes selected using the CytoHubba plugin according to the MCC method are sequentially ordered in Table 1(b). The PPI network was divided into two clusters (clusters A and B) using the MCODE plugin (Figures 4(a) and 4(b)). Interestingly, most of the top 40 genes identified using the different calculation methods, previously mentioned herein, were present in both clusters A and B, with the genes shown in Table 1(a) being in cluster A and genes in Table 1(b) in cluster B. These findings suggested that these genes play important roles in sepsis. Tables 2(a) and 2(b) show the functional annotation of the two clusters. Cluster A was enriched in genes involved in T-cell biology, antigen presentation, and natural killer (NK) cell function. Intriguingly, most genes in cluster A were downregulated, suggesting a possible immunosuppression process early in sepsis. In contrast, cluster B mainly contained genes related to innate immune responses such as neutrophil-mediated immunity and phagocytosis. These genes are closely related and cooperate with each other to respond to different types of insults. Overall, these 40 genes comprised the hub genes during sepsis. While some of these genes are well-characterized key elements in sepsis, others might represent new potential biomarkers for sepsis. Nine genes (*LRG1*, *ELANE*, *TP53*, *LCK*, *TBX21*, *ZAP70*, *CD247*, *ITK*, and *FYN*) were chosen for further investigation of their roles in and potential use as biomarkers for sepsis.

### 3.4. ROC Curve

**Figure 5:**
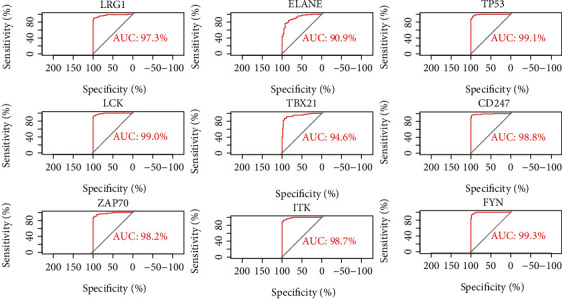
ROC curve analyses of 9 selected DEGs.

To identify new potential biomarkers for sepsis, ROC curves of data derived from healthy controls and patients with sepsis from datasets GSE57065, GSE95233, and GSE28750 were analyzed using the R package (Figure 5). ROC curves were generated, and the area under the curves was used to compare the different genes. This analysis demonstrated that the nine selected genes had a diagnostic role in sepsis. Thus, we chose these genes as candidates for further analysis and validation.

### 3.5. Validation of Selected Genes at the Transcriptional Level

**Figure 6:**
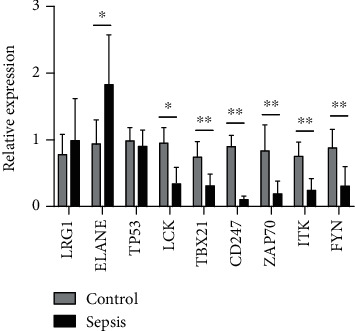
Expression of nine selected key genes was compared between healthy controls and sepsis patients by quantitative real-time PCR. Differences between two groups were analyzed using the *t*-test. ^∗^*P* < 0.05, ^∗∗^*P* < 0.01.

The expression of nine key genes was compared between patients with sepsis (*n* = 5) and healthy controls (*n* = 5) using quantitative real-time PCR. The results showed that the expression of seven of these genes was consistent with the trend observed in the microarray analysis, whereas two genes, LRG1 and TP53, showed no significant difference in expression (Figure 6).

## 4. Discussion

A microarray study is an ideal way to comprehensively investigate sepsis. In this study, three gene datasets were integrated to search for potential biomarkers and explore molecular mechanisms of sepsis. Although sepsis is an inflammatory disease, it has recently been established that both proinflammatory and anti-inflammatory responses occur early during sepsis [10]. In our study, genes associated with both innate and adaptive immunity had altered expression patterns in patients with sepsis since the beginning of diagnosis. KEGG pathway enrichment analysis showed that different antigenic constituents from bacteria, viruses, and other insults can cause sepsis. Upon receptor contact with their cognate ligands, proinflammatory intermediates are recruited and intracellular signaling pathways such as NF-*κ*B transduction are activated. The activation of NF-*κ*B induces the expression of early activation genes such as *IL1B*, *IFNG*, and *IL*-*6* to combat the insult. However, excessive cytokine release leads to an increase in the release of circulating immune cells [5]. We found that most genes in cluster B encoded proteins that participate in innate immune responses. All of these genes were upregulated, indicating their roles in innate immune responses. One of these genes is associated with OLFM4+ neutrophils, a subset of human neutrophils. Elevated levels of OLFM4+ neutrophils in patients with sepsis are associated with worse outcomes [11]. CHI3L1 is produced by several cells including macrophages and neutrophils. A recent study pointed out that the downregulation of CHI3L1 alleviates skeletal muscle stem cell injury, suggesting its therapeutic potential for sepsis [12]. In our study, we found that the genes in cluster B interact with one another to mediate inflammatory responses. Targeting these genes and corresponding pathways involved in innate immune responses might be one strategy to reduce inflammation and associated pathology during sepsis, although much work is still required, considering the previous unsatisfactory trials involving immune activation genes.

Previously, it was thought that the host immune response to sepsis is characterized by an initial hyperinflammatory response, followed by an immunosuppressive phase as the disease progresses. However, recent studies have shown that both proinflammatory and anti-inflammatory responses occur early and simultaneously in sepsis [13]. In our study, genes in cluster A were found to be mainly involved in T-cell biology, antigen processing and presentation, and NK cell function. Interestingly, all genes in cluster A were downregulated, suggesting immunosuppression during sepsis. In cluster A, genes encoding antigen presentation-related molecules, including HLA-DRA, HLA-DRB1, HLA-DPA1, HLA-DPB1, and CCR7, exhibited decreased expression. Studies have also shown that during sepsis, the number of dendritic cells (DCs), the major group associated with antigen presentation, is decreased in patients with sepsis. In addition, the surviving DCs also exhibit lower expression of HLA-DR. Moreover, endotoxin-tolerant macrophages express relatively low levels of HLA-DR on their surface, resulting in a lack of antigen presentation [14]. Similarly, NK cells are also underrepresented in patients with sepsis, and the remaining NK cells display defective cytotoxic functions [15, 16]. In our study, we found that genes involved in NK cell-mediated cytotoxicity (such as *KLRB1*, *KLRD1*, *SH2D1A*, and *PRF1*) showed decreased expression. In cluster A, *LCK*, *ZAP70*, *CD2*, *CD247*, *CD27*, *CD28*, *CD3E*, *CD3G*, *CD4*, *CD8A*, *CD8B*, *ITK*, *LCK*, *TRAT1*, *TBX21*, *FYN*, and *IL*-*7R*, which are related to T-cell biology, were represented, suggesting an important role for T-cell immunity during sepsis. Recently, the immunosuppressive phase has become a focus during sepsis treatment. It is inspiring that IL-7, a growth factor that stimulates the proliferation and maturation of many cell types, could continuously boost the absolute T lymphocyte counts of sepsis patients in a phase II clinical trial [17]. Our results also indicated that immunostimulatory therapy targeting immunosuppression-related genes and pathways could be a promising way to treat sepsis.

It should be noted that there were some DEGs that were not shared among all sepsis networks but have been proven to play indispensable roles in sepsis in recent years. For example, RETN encodes resistin, which is strikingly elevated in patients with sepsis and is associated with sepsis severity and outcomes [18]. Silswal et al. showed that resistin activated monocytes and macrophages as well as induced the release of proinflammatory cytokines [19]. Further, recent experimental data have highlighted resistin as a potential therapeutic target in sepsis [20]. These previous findings suggest a paradoxical role for resistin in sepsis. TCN1 encodes a member of the vitamin B_12_-binding protein family, transcobalamin I, which is elevated during infectious conditions. As a member of the cobalamin transport protein, transcobalamin elevation may contribute to the resolution of inflammation [21]. FOLR3 and GGH are associated with the folate pathway. These reports suggest that vitamin B_12_ and folate metabolism may also constitute an important part of sepsis. Nutritional therapy, including vitamin B_12_ and folate, may affect the pathogenesis of sepsis, which requires further research [22].

In this study, we also identified some genes for which the functions in sepsis have not been completely characterized, suggesting their potential as biomarkers for this disease. It should be noted that the expression levels of most of these genes demonstrated by real-time PCR corresponded to the patterns observed by microarray analyses, while two genes (LRG1, TP53) showed no significant difference. The inconsistency could be attributed to the different detection methods, sample size, patient heterogeneity, and course of the disease. Considering the small sample size of our study, it is not powerful enough to change the conclusions about the nine critical genes selected by bioinformatics analysis.

One of these genes, *LRG1*, encodes a highly conserved member of the leucine-rich repeat family of proteins, which has been reported to play a role in the inflammatory response. LRG1 is expressed by neutrophils and macrophages [23, 24]. Some studies found that circulating *LRG1* mRNA and plasma LRG1 protein levels might together be helpful for diagnosing simple and complicated acute appendicitis in patients with acute abdominal pain [25]. However, the role of LRG1 in sepsis remains unclear to the best of our knowledge. Our microarray analyses showed that *LRG1* mRNA levels are higher in patients with sepsis. Further study is needed to validate these results and investigate the roles of this marker in sepsis.

Another gene, *ELANE*, encodes neutrophil elastase (NE), a serine protease secreted by neutrophils into the extracellular milieu during the inflammatory response [26]. NE also participates in the formation of neutrophil extracellular traps [27]. A previous study found that NE is positively correlated with the severity of sepsis and organ dysfunction, suggesting its potential as a biomarker for sepsis [28]. This finding is consistent with our results.

LCK and FYN belong to the Src family of protein tyrosine kinases. LCK phosphorylates downstream signaling proteins, resulting in changes in the expression of genes that are essential for T-cell maturation and activation. FYN is involved in signal transduction pathways during the development and activation of T lymphocytes and NK cells under physiological conditions [29]. FYN and LCK are also essential for platelet production and activation [30]. However, the roles of LCK and FYN in sepsis have not been entirely elucidated. In our study, the expression of LCK and FYN was decreased in patients with sepsis, indicating possible suppression of T-cells and NK cells, as well as a role for these proteins in platelet functions during sepsis, which warrants further exploration. Our ROC curve analysis also showed that both genes have diagnostic value for sepsis.

ZAP70, a member of the Syk protein kinase family, is enriched in the TCR signaling pathway. This protein functions in the initial step of TCR-mediated signal transduction in combination with the Src family kinases LCK and FYN. Functional deletion of ZAP70 can lead to selective T-cell defects characterized by the selective absence of CD8-positive T-cells. Gomez-Rodriguez et al. [31] found that the downregulation of ZAP70 accelerates neonatal sepsis disease progression. The role of ZAP70 in adult sepsis warrants further investigation.

The gene product of CD247 (also known as CD3*ζ*) is T-cell receptor zeta, which is part of the T-cell receptor-CD3 complex. CD247 plays essential roles in coupling antigen recognition to several intracellular signal transduction pathways. CD3*ζ* chain expression is consistently reduced in T-cells from both the spleen and lymph nodes in sepsis [32]. Moreover, the downregulation of CD247 was reported to be accompanied by decreased expression of other T-cell-associated signal transduction molecules such as ZAP70, as well as T-cell apoptosis, in line with our data.

TP53 is a well-characterized gene for which the gene product induces cell cycle arrest, apoptosis, senescence, DNA repair, and metabolic changes [32]. TP53 may contribute to apoptosis in a tissue-dependent manner. A recent study revealed that p53 expression in T lymphocytes during sepsis could be responsible for enhancing both apoptosis and immune dysfunction in T-cells [33]. In our microarray analyses, TP53 expression was found to be downregulated in patients with sepsis, which is in accordance with the expression of genes involved in the T-cell signaling pathway, suggesting their possible interaction during sepsis.

TBX21, also known as T-bet, is the master regulator of effector T-cell activation. Many studies have shown that TBX21 controls the expression of IFNG, a hallmark Th1 cytokine, suggesting a role for this protein in initiating Th1 lineage development [34]. Recently, studies have also discovered T-bet expression in B-cells, CD8+ T-cells, and T-reg cells, suggesting variable functions under different circumstances [35–37]. The decrease in TBX21 expression might influence the expression of related immune cells, a notion that warrants further study.

Finally, ITK, which belongs to the Tec tyrosine kinase family, is involved in multiple aspects of T-cell development and functions such as T-cell activation and T-helper cell differentiation [31]. ITK is also known to be involved in the development of Th17 cells by regulating various transcription factors. For example, in ALI mice, ITK regulates the balance between inflammatory Th17 cells and anti-inflammatory T-reg cells [38]. However, the role of ITK in T-cell functions in patients with sepsis has not been fully evaluated. Our study showed that ITK was downregulated in patients, although further research is needed to explore the underlying mechanism.

## 5. Conclusions

Using a bioinformatics analysis of three gene datasets (GSE95233, GSE57065, and GSE28750), we identified the immune characteristics of sepsis. We found that DEGs in patients were enriched for pathways mainly involved in the innate immune response, T-cell biology, antigen presentation, and NK cell function. Focusing on the key genes and corresponding pathways involved in sepsis could provide new insights for sepsis treatment. Nine genes including *LRG1*, *ELANE*, *TP53*, *LCK*, *TBX21*, *ZAP70*, *CD247*, *ITK*, and *FYN* were also identified as potential new biomarkers for sepsis. The expression levels of most of these genes demonstrated by real-time PCR corresponded to the patterns observed by microarray analyses. Further investigations are needed to validate these preliminary findings.

## Figures and Tables

**Table 1a:** (a) The top 20 genes in the network ranked by degrees

Name	Degree	Change	Cluster
TP53	76	Down	
LCK	59	Down	1
MMP9	56	Up	2
CCL5	54	Down	1
CD28	53	Down	1
TBX21	50	Down	1
CD2	48	Down	1
ZAP70	48	Down	1
CD3E	46	Down	1
CCR7	46	Down	1
CD4	44	Down	1
MPO	43	Up	
IL2RB	43	Down	1
CD247	42	Down	1
ELANE	42	Up	1; 2
ITK	42	Down	1
GATA3	41	Down	1
C3AR1	41	Up	1
PRF1	40	Down	1
FYN	40	Down	1

**Table 1b:** (b) The top 20 genes in the network ranked by maximal clique centrality (MCC)

Name	MCC	Change	Cluster
ELANE	9.22*E* + 13	Up	1; 2
SLPI	9.22*E* + 13	Up	2
HP	9.22*E* + 13	Up	2
PTX3	9.22*E* + 13	Up	2
DEFA4	9.22*E* + 13	Up	2
BPI	9.22*E* + 13	Up	2
CAMP	9.22*E* + 13	Up	2
ORM1	9.22*E* + 13	Up	2
LCN2	9.22*E* + 13	Up	2
MMP9	9.22*E* + 13	Up	2
PGLYRP1	9.22*E* + 13	Up	2
RAB27A	9.22*E* + 13	Up	2
MMP8	9.22*E* + 13	Up	2
LTF	9.22*E* + 13	Up	2
TCN1	9.22*E* + 13	Up	2
LRG1	9.22*E* + 13	Up	2
RETN	9.22*E* + 13	Up	2
ARG1	9.22*E* + 13	Up	2
OLFM4	9.22*E* + 13	Up	2
CHI3L1	9.22*E* + 13	Down	2

**Table 2a:** (a) Functions enriched for the genes involved in cluster A

Term	Description	Count	*P* value
GO:0050852	T-cell receptor signaling pathway	16	1.92*E* − 23
GO:0006955	Immune response	14	1.76*E* − 13
GO:0031295	T-cell costimulation	12	8.13*E* − 19
GO:0007166	Cell surface receptor signaling pathway	11	3.72*E* − 11
GO:0007169	Transmembrane receptor protein tyrosine kinase signaling pathway	8	3.76*E* − 10
GO:0050776	Regulation of immune response	8	2.83*E* − 08
GO:0042110	T-cell activation	7	2.41*E* − 10
GO:0019882	Antigen processing and presentation	7	6.44*E* − 10
GO:0042102	Positive regulation of T-cell proliferation	7	1.11*E* − 09
GO:0002504	Antigen processing and presentation of peptide or polysaccharide antigen via MHC class II	6	9.31*E* − 11
GO:0060333	Interferon-gamma-mediated signaling pathway	6	1.83*E* − 07
GO:0019886	Antigen processing and presentation of exogenous peptide antigen via MHC class II	6	6.72*E* − 07
GO:0002250	Adaptive immune response	6	7.02*E* − 06
GO:0045087	Innate immune response	6	0.00105484
hsa04660	T-cell receptor signaling pathway	11	2.82*E* − 12
hsa04514	Cell adhesion molecules (CAMs)	11	9.77*E* − 11
hsa04612	Antigen processing and presentation	10	8.8*E* − 12
hsa05166	HTLV-1 infection	10	4.13*E* − 07
hsa05332	Graft-versus-host disease	9	3.27*E* − 13
hsa05330	Allograft rejection	9	8.99*E* − 13
hsa04940	Type I diabetes mellitus	9	2.71*E* − 12
hsa05320	Autoimmune thyroid disease	9	1.68*E* − 11
hsa04640	Hematopoietic cell lineage	9	1.19*E* − 09
hsa05416	Viral myocarditis	8	1.9*E* − 09
hsa05323	Rheumatoid arthritis	8	4.19*E* − 08
hsa05340	Primary immunodeficiency	7	3.19*E* − 09
hsa04672	Intestinal immune network for IgA production	7	2.46*E* − 08
hsa05321	Inflammatory bowel disease (IBD)	7	1.64*E* − 07
hsa04650	Natural killer cell-mediated cytotoxicity	7	0.00000753
hsa05322	Systemic lupus erythematosus	7	0.0000129

**Table 2b:** (b) Functions enriched for the genes involved in cluster B

Term	Description	Count	*P* value
GO:0045087	Innate immune response	7	1.90*E* − 05
GO:0006508	Proteolysis	5	0.004385255
GO:0019731	Antibacterial humoral response	4	2.87*E* − 05
GO:0006955	Immune response	4	0.019097644
GO:0001878	Response to yeast	3	1.39*E* − 04
GO:0050829	Defense response to Gram-negative bacterium	3	0.002549924
GO:0071347	Cellular response to interleukin-1	3	0.004210679
GO:0022617	Extracellular matrix disassembly	3	0.004809135
GO:0050830	Defense response to Gram-positive bacterium	3	0.005979232
GO:0071356	Cellular response to tumor necrosis factor	3	0.009834724
GO:0071222	Cellular response to lipopolysaccharide	3	0.010355235
GO:0045766	Positive regulation of angiogenesis	3	0.010708943
GO:0042742	Defense response to bacterium	3	0.016637646
GO:0006032	Chitin catabolic process	2	0.009550291
GO:0009635	Response to herbicide	2	0.009550291
GO:0019732	Antifungal humoral response	2	0.01361651
GO:0044130	Negative regulation of growth of symbiont in host	2	0.021701096
GO:0070207	Protein homotrimerization	2	0.029722278
GO:0002227	Innate immune response in mucosa	2	0.033709241
GO:0035987	Endodermal cell differentiation	2	0.036358505
GO:0032715	Negative regulation of interleukin-6 production	2	0.03768053
GO:0050766	Positive regulation of phagocytosis	2	0.03900082
GO:0010043	Response to zinc ion	2	0.048194457

## Data Availability

The data used to support the findings of this study are available from the corresponding authors upon request.

## Supplementary Materials

Supplementary Materials Supplementary data 1: primers of 10 genes.
