# Supplementary material for: Identification of Potential Biomarkers and Immune Features of Sepsis Using Bioinformatics Analysis
Source: Mediators Inflamm. 2020 Oct 9;2020:3432587. doi: 10.1155/2020/3432587 (PMC7568774; doi:10.1155/2020/3432587)
Supplement: Supplementary Materials — Supplementary data 1: primers of 10 genes. [file 3432587.f1.docx]

Supplementary data1. Primers of 10 genes

| Gene | Genbank Accession | Primer Sequences(5'to3') |
| --- | --- | --- |
| Human GAPDH | NM_002046.5 | CCATGACAACTTTGGTATCGTGGAA |
|  |  | GGCCATCACGCCACAGTTTC |
| Human P53 | NM_001126118.1 | GGAACAGCTTTGAGGTGCGTGTTT |
|  |  | CCCCTTTCTTGCGGAGATTCTCT |
| Human ELANE | NM_001972.4 | GCACTGCGTGGCGAATGTA |
|  |  | GGGTCGTAGCCGTTTTCG |
| Human LCK | NM_001042771.3 | CATGGAGAATGGGAGTCTAGTG |
|  |  | GCCATGTCCAGGAGTTTGTTGAT |
| Human TBX21 | NM_013351.2 | GCTCCAGTCCCTCCATAAGTA |
|  |  | GCGTGTTGGAAGCGTTGCA |
| Human ZAP70 | NM_001079.4 | CCACCCATCCACGTTGACT |
|  |  | GGTTTGTCTGGGGACGTTATG |
| Human CD247 | NM_000734.4 | GCCTGTACAATGAACTGCAGAAAG |
|  |  | CGCTCGCCTTTCATCCCAAT |
| Human ITK | NM_005546.4 | CCCTCAGAGCTCACTTTTGTG |
|  |  | CTTGTCCTTGTTGAGCCAGTAG |
| Human FYN | XM_005266892.4 | CACAGCAAGACAAGGTGCAAAG |
|  |  | CACGTCAGACTTGATTGTGAACCT |
| Human LRG1 | NM_052972.3 | GGAGAACCAGTTGGAGACCTT |
|  |  | GCCTTCTAGATGTAGCCGTTCT |
